# 1360. Investigating Transmission Dynamics Between Pets and Caretakers in Households of Children with Methicillin-resistant *S. aureus* Skin and Soft Tissue Infections

**DOI:** 10.1093/ofid/ofac492.1189

**Published:** 2022-12-15

**Authors:** Hannah Kinzer, Sara Malone, Mary G Boyle, Lauren Norton, Rinat Tal, Hunter Olson, Stephanie A Fritz

**Affiliations:** Washington University in St. Louis, St. Louis, Missouri; Washington University School of Medicine, Saint Louis, Missouri; Washington University School of Medicine, Saint Louis, Missouri; Washington University School of Medicine, Saint Louis, Missouri; Washington University School of Medicine, Saint Louis, Missouri; Washington University School of Medicine, Saint Louis, Missouri; Washington University School of Medicine, Saint Louis, Missouri

## Abstract

**Background:**

Household pets are susceptible to *Staphylococcus aureus* (*S. aureus*) colonization. Our objective was to examine transmission of *S. aureus* between pets and their caretakers following a human household decolonization intervention.

**Methods:**

From 2015-2021, a pragmatic randomized trial enrolled 196 households of children with community-associated methicillin-resistant *S. aureus* skin and soft tissue infection. Mouth and dorsal fur samples from indoor dogs and cats were cultured. Human nares, axillae, and inguinal fold samples were cultured. Samples were collected at enrollment and 1, 3, 6, and 9 months post-intervention. Participants were asked about pet daycare use, whether pets slept with caretakers, and pet skin conditions. Univariate and bivariate analyses, including McNemar tests, were conducted. Inclusion criteria were cat or dogs with at least one swab pre- and post-intervention.

**Results:**

Our populations included 95 households with 161 pets; 35 households (39%) had ≥1 pet colonized with *S. aureus*. Within 2 weeks prior to enrollment, 3 (2%) pets had attended daycare, 70 (44%) slept with a caretaker, and 16 (10%) had skin conditions (see Table 1). 27 (17%) pets were colonized at enrollment. Of the 121 dogs, 20 (17%) were colonized at enrollment, 9 (7%) with MRSA. Of the 40 cats, 7 (18%) were colonized at enrollment, and 4 (10%) were colonized with MRSA. 72 (45%) pets had ≥1 caretakers who were colonized at enrollment; 18 (25%) of these pets were also colonized. Of the 137 caretakers, 48 (35%) were colonized at enrollment, and 16 (12%) were colonized with MRSA.

Whether a pet was colonized at enrollment was associated with colonization post-intervention (p< 0.001, see Table 2). All pets that were colonized at enrollment remained colonized post-intervention. Caretaker colonization at enrollment was associated with pet colonization post-intervention (p=0.04). Of the 72 pets whose caretakers were colonized at baseline, 36 (50%) pets were colonized post-intervention.

**Figure:**
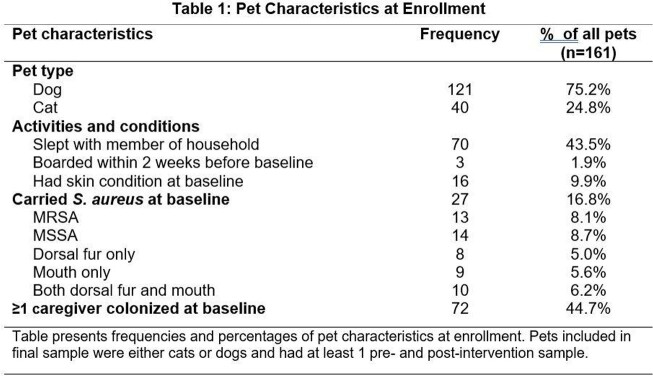

**Figure:**
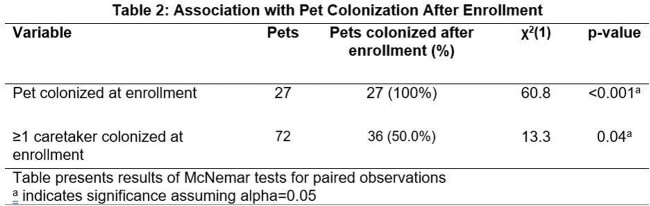

**Conclusion:**

Both pet colonization and caretaker colonization at enrollment are significantly associated with pet colonization after human household member decolonization. This indicates that *S. aureus* may transmit from human caretakers to pets and human decolonization may not be fully protective against pet colonization.

**Disclosures:**

**Sara Malone, PhD, LCSW**, AHRQ: Grant/Research Support|NIH: Grant/Research Support.

